# Supplementary material for: From sequence to enzyme mechanism using multi-label machine learning
Source: BMC Bioinformatics. 2014 May 19;15:150. doi: 10.1186/1471-2105-15-150 (PMC4229970; doi:10.1186/1471-2105-15-150)
Supplement: Additional file 2 — Java code of ml2db. Additional file ml2db_code.tar.gz contains the Java source code to run the multi-label machine learning experiments and save the results to database. The code’s Javadoc is included. [file 1471-2105-15-150-S2.zip › additional file 2/ml2db/ml2db/doc/main/ml2db/weka/InstAttValueMap.html]

InstAttValueMap


JavaScript is disabled on your browser.


- Overview
- Package
- Class
- Use
- Tree
- Deprecated
- Index
- Help

- Prev Class
- Next Class

- Frames
- No Frames

- All Classes

- Summary:
- Nested |
- Field |
- Constr |
- Method

- Detail:
- Field |
- Constr |
- Method


main.ml2db.weka

## Class InstAttValueMap

- java.lang.Object
- - java.util.AbstractMap<K,V>
  - - java.util.TreeMap<java.lang.String,java.util.Map<java.lang.String,java.lang.String>>
    - - uk.ac.standrews.utils.main.maputils.MapOfMaps
      - - main.ml2db.weka.InstAttValueMap

- All Implemented Interfaces:
  :   java.io.Serializable, java.lang.Cloneable, java.util.Map<java.lang.String,java.util.Map<java.lang.String,java.lang.String>>, java.util.NavigableMap<java.lang.String,java.util.Map<java.lang.String,java.lang.String>>, java.util.SortedMap<java.lang.String,java.util.Map<java.lang.String,java.lang.String>>

  ---

    

  ```
  public class InstAttValueMap
  extends uk.ac.standrews.utils.main.maputils.MapOfMaps
  ```

  Contains [instance identifier, attribute name, attribute value] tuples. The
  map must accept empty strings as attribute/value: otherwise empty instances
  (without any attribute or class value) are not represented and cannot be
  created. However, empty attribute names ("") should not appear in the list of
  attribute names.

  Version:
  :   27 Feb 2013

  Author:
  :   Luna De Ferrari ldf(at)st-andrews.ac.uk or
      luna.deferrari@st-andrews.ac.uk

  See Also:
  :   Serialized Form

- - ### Nested Class Summary

    - ### Nested classes/interfaces inherited from class java.util.AbstractMap

      `java.util.AbstractMap.SimpleEntry<K,V>, java.util.AbstractMap.SimpleImmutableEntry<K,V>`
  - ### Field Summary

    Fields

    | Modifier and Type | Field and Description |
    | --- | --- |
    | `static java.lang.String` | `CLASS_ATTRIBUTES_TAG` |
    | `static java.lang.String` | `FEATURE_ATTRIBUTES_TAG` |
  - ### Constructor Summary

    Constructors

    Constructor and Description

    `InstAttValueMap(boolean acceptEmptyStrings,
    java.lang.String feature_or_class_attributes)`

    `InstAttValueMap(boolean acceptEmptyStrings,
    java.util.Vector<java.lang.String> extKeys,
    java.util.Vector<java.lang.String> intKeys,
    java.util.Vector<java.lang.String> intValues,
    java.lang.String feature_or_class_attributes)`
  - ### Method Summary

    Methods

    | Modifier and Type | Method and Description |
    | --- | --- |
    | `void` | `addAll(uk.ac.standrews.utils.main.maputils.MapOfMaps anotherMap)` Sets the type of attribute after adding data |
    | `java.util.TreeSet<java.lang.String>` | `getAttributeNames()` |
    | `java.util.Map<java.lang.String,java.lang.String>` | `getAttributesValues(java.lang.String instanceName)` |
    | `java.lang.String` | `getNeedsNominalOrNumeric()` |
    | `int` | `getNumberOfAttributes()` Get how many attributes have a value (any value) for any instance in the map |
    | `int` | `getNumberOfInstanceAttributeCouples()` |
    | `java.lang.String` | `guessNeedsNominalOrNumeric()` |
    | `void` | `setNeedsNominalOrNumeric(java.lang.String attributeType)` |

    - ### Methods inherited from class uk.ac.standrews.utils.main.maputils.MapOfMaps

      `acceptEmptyStrings, addKeyKeyValue, containsInternalKeyValue, containsKeyKeyValue, countInternalKeys, generateInternalKeys, getExternalInternalKeyMap, getExtStringPool, getInternalKeys, getIntStringPool, getUniqueValues, getValuesStringPool, toString`
    - ### Methods inherited from class java.util.TreeMap

      `ceilingEntry, ceilingKey, clear, clone, comparator, containsKey, containsValue, descendingKeySet, descendingMap, entrySet, firstEntry, firstKey, floorEntry, floorKey, get, headMap, headMap, higherEntry, higherKey, keySet, lastEntry, lastKey, lowerEntry, lowerKey, navigableKeySet, pollFirstEntry, pollLastEntry, put, putAll, remove, size, subMap, subMap, tailMap, tailMap, values`
    - ### Methods inherited from class java.util.AbstractMap

      `equals, hashCode, isEmpty`
    - ### Methods inherited from class java.lang.Object

      `getClass, notify, notifyAll, wait, wait, wait`
    - ### Methods inherited from interface java.util.Map

      `equals, hashCode, isEmpty`

- - ### Field Detail


    - #### FEATURE\_ATTRIBUTES\_TAG

      ```
      public static final java.lang.String FEATURE_ATTRIBUTES_TAG
      ```

      See Also:
      :   Constant Field Values


    - #### CLASS\_ATTRIBUTES\_TAG

      ```
      public static final java.lang.String CLASS_ATTRIBUTES_TAG
      ```

      See Also:
      :   Constant Field Values
  - ### Constructor Detail


    - #### InstAttValueMap

      ```
      public InstAttValueMap(boolean acceptEmptyStrings,
                     java.lang.String feature_or_class_attributes)
      ```


    - #### InstAttValueMap

      ```
      public InstAttValueMap(boolean acceptEmptyStrings,
                     java.util.Vector<java.lang.String> extKeys,
                     java.util.Vector<java.lang.String> intKeys,
                     java.util.Vector<java.lang.String> intValues,
                     java.lang.String feature_or_class_attributes)
      ```

      Parameters:
      :   `acceptEmptyStrings` -
      :   `extKeys` -
      :   `intKeys` -
      :   `intValues` -
      :   `feature_or_class_attributes` - whether the map will contain class of feature attributes. A
          different string will be prepended to the attribute name
          depending on this.
  - ### Method Detail


    - #### addAll

      ```
      public void addAll(uk.ac.standrews.utils.main.maputils.MapOfMaps anotherMap)
      ```

      Sets the type of attribute after adding data

      **Overrides:**
      :   `addAll` in class `uk.ac.standrews.utils.main.maputils.MapOfMaps`


    - #### getAttributeNames

      ```
      public java.util.TreeSet<java.lang.String> getAttributeNames()
      ```


    - #### getAttributesValues

      ```
      public java.util.Map<java.lang.String,java.lang.String> getAttributesValues(java.lang.String instanceName)
      ```


    - #### getNeedsNominalOrNumeric

      ```
      public java.lang.String getNeedsNominalOrNumeric()
      ```


    - #### getNumberOfAttributes

      ```
      public int getNumberOfAttributes()
      ```

      Get how many attributes have a value (any value) for any instance in the
      map

      Returns:


    - #### getNumberOfInstanceAttributeCouples

      ```
      public int getNumberOfInstanceAttributeCouples()
      ```


    - #### guessNeedsNominalOrNumeric

      ```
      public java.lang.String guessNeedsNominalOrNumeric()
      ```

      Returns:
      :   the type of attributes needed depending on the data in the map
          (nominal or numeric)


    - #### setNeedsNominalOrNumeric

      ```
      public void setNeedsNominalOrNumeric(java.lang.String attributeType)
      ```


- Overview
- Package
- Class
- Use
- Tree
- Deprecated
- Index
- Help

- Prev Class
- Next Class

- Frames
- No Frames

- All Classes

- Summary:
- Nested |
- Field |
- Constr |
- Method

- Detail:
- Field |
- Constr |
- Method
